# Book and Pamphlet Notices

**Published:** 1859-12

**Authors:** 


### BOOK AND PAMPHLET NOTICES.

A System of Surgery—Pathological, Diagnostic, Therapeutic and Operative. By Samuel Gross, M.D., Prof. of Surgery in Jefferson Medical College, etc. etc. Illustrated by 936 Engravings. In two volumes. Pp. 1162 and 1196. Philadelphia: Lea & Blanchard. 1859.

We have deferred the notice of this voluminous work of Prof. Gross in order to be able to speak with better knowledge of its value, but confess that up to the present time we have not been able to do more than examine some of its more important chapters. Indeed the perusal of 2358 pages, the greater part of which is necessarily made up of compilation from various sources, is a task to which we feel but little disposed, more as the merits and defects of the author are sufficiently known from his former works.

Notwithstanding the great extent of this work, it were easy to point out defects as to completeness. The pathology it inculcates is, in many instances, contrary to our own views, as well as to those generally believed. Many of the illustrations are familiar to the profession from having been used in other volumes. The subject is much too vast to be fully treated in a book even so ponderous as this; and when the chapters on pathology are compared with the work of Paget, those on fractures with that of Malgaigne, on operations on the eye with that of Walton, etc., the advantage of these monographs is very apparent. These, however, are difficulties which no writer could overcome. When Prof. Gross speaks of his own experience and gives first illustrations, his work is throughout interesting and readable. The labor of its preparation was so great that we cannot but admire the courage which would prompt the undertaking. Without presuming to compare it with such works on surgery as that of Boyce in French, or Coope's Surgical Dictionary in English, we can confidently recommend it as a useful treatise for reference, and a valuable and ever necessary addition to every surgical library.

